# An imputation method for single‐cell RNA sequencing data based on a parallel gated recurrent unit neural network

**DOI:** 10.1002/qub2.70054

**Published:** 2026-09-29

**Authors:** Shuang Xu, Xiangtao Li, Jingsong Li, Yu Jiang

**Affiliations:** ^1^ Department of Anesthesiology The Second Hospital of Jilin University Changchun China; ^2^ College of Software Jilin University Changchun China; ^3^ College of Computer Science and Technology Jilin University Changchun China

**Keywords:** clustering models, gated recurrent unit, imputation, prediction algorithm, recurrent neural network, single‐cell RNA sequence

## Abstract

Single‐cell RNA sequencing (scRNA‐seq) generates high‐dimensional transcriptomic data but is severely affected by dropout events, which obscure true gene–gene relationships. These missing values introduce bias into downstream analyses, complicate data processing, and reduce analytical efficiency. Therefore, accurate imputation of dropout events is essential for recovering genuine expression signals and improving the reliability of scRNA‐seq studies. In this work, we propose a novel scRNA‐seq data imputation method, imputation with a parallel gated recurrent unit neural network (IPGRU), based on a parallel gated recurrent unit (GRU) neural network. IPGRU reformulates the original multifeature prediction problem into multiple single‐feature prediction tasks, thereby substantially reducing the complexity of the imputation process. By exploiting dependencies between expressed and missing genes, the GRU hidden states are employed to model gene expression patterns and predict dropout events, enabling effective correction of missing values and improving data completeness. Experimental results demonstrate that IPGRU achieves high accuracy in imputing dropout events and significantly enhances the integrity of scRNA‐seq data. Moreover, the imputed data provide more reliable inputs for downstream analyses, including cell clustering and inference of differentiation trajectories. IPGRU offers a robust and efficient solution for scRNA‐seq data imputation, improving the accuracy and reliability of downstream biological analyses. This method has strong potential to facilitate biological discovery and advance precision medicine.

## 1 INTRODUCTION

Traditional bulk RNA sequencing methods capture only the average gene expression levels across heterogeneous cell populations, thereby obscuring cell‐to‐cell variability and masking critical biological features. As a result, accurately characterizing gene expression differences at the single‐cell level remains challenging. In contrast, single‐cell RNA sequencing (scRNA‐seq) enables the measurement of gene expression profiles at single‐cell resolution and has fundamentally transformed our understanding of diseases and associated biological processes by revealing cellular heterogeneity within complex tissues [1, 2, 3, 4]. Consequently, single‐cell‐based life science research provides a more fundamental and mechanistic basis for investigating the etiology, progression, and treatment of major diseases.

By overcoming the limitations of bulk sequencing, scRNA‐seq captures transcriptional variability within cell populations and enables more precise cell classification and characterization [5, 6]. However, scRNA‐seq data are inherently affected by technical limitations, including missingness, high noise levels, and sparsity, primarily due to the limited amounts of RNA obtained from individual cells and the shallow sequencing depth [7, 8, 9].

Zero expression values in scRNA‐seq data, arising from inefficient RNA capture and amplification, low endogenous gene expression, or stochastic transcriptional dropout, are commonly referred to as dropout events [7, 10]. A high prevalence of dropout events severely compromises downstream analyses, distorting gene–gene relationships and impairing cell‐type identification in clustering tasks [11]. Therefore, effective restoration of true gene expression patterns through advanced imputation strategies, particularly those based on deep learning, is essential for improving the quality and reliability of scRNA‐seq data analysis.

Restoring scRNA‐seq data and improving data quality are critical for enhancing the efficiency and reliability of downstream gene expression analyses. One of the most important strategies for addressing zero values in scRNA‐seq data is imputation, which infers or replaces missing expression values based on observed data patterns [12, 13, 14]. Over the past decade, numerous imputation methods have been developed to mitigate the impact of dropout events in scRNA‐seq datasets.

Early approaches include the LATE model proposed by Badsha et al. in 2016, which performed gene expression recovery for small‐scale datasets by extracting original gene features and leveraging transfer learning strategies [15, 16]. In 2018, Van Dijk et al. introduced Markov affinity‐based graph imputation of cells (MAGIC) [17], which uses data diffusion on a cell‐similarity graph to denoise expression matrices and recover missing transcripts. Li et al. proposed scImpute, a statistically driven method that automatically identifies potential dropout events and selectively imputes zero values while avoiding biasing nonzero entries [18].

With the advancement of deep learning, Arisdakessian et al. first applied neural networks to scRNA‐seq imputation and proposed DeepImpute in 2019 [19], demonstrating superior robustness compared with traditional statistical approaches. Talwar et al. developed AutoImpute [20], an autoencoder‐based sparse interpolation method that minimizes perturbations to biologically silent genes. Subsequently, Eraslan et al. improved autoencoder architectures and proposed the deep count autoencoder (DCA), a three‐layer output model capable of capturing complex nonlinear dependencies in scRNA‐seq data [21]. Zhu et al. further introduced 2DImpute, which predicts false‐zero values by jointly modeling gene–gene and cell–cell relationships to prevent overcorrection [22]. Graph neural network (GNN)‐based approaches, such as scGNN, have demonstrated strong performance by modeling cell–cell relationships through graph structures [23].

In bioinformatics, recurrent neural networks (RNNs) [24], particularly gated recurrent units (GRUs) [25] and long short‐term memory (LSTM) networks [26], have been widely adopted for modeling sequential data, such as gene expression profiles and biological sequences. Ozsahin et al. [27] utilized bidirectional LSTM and GRU architectures to predict epitopes from severe acute respiratory syndrome coronavirus 2 (SARS‐CoV‐2) proteins using a bioinspired optimization framework. Han et al. [28] proposed SEOE, a graph‐based representation method integrating word2vec [29], LSTM, and GRU for survey questionnaire analysis, achieving high accuracy in depression screening. Chai et al. [30] developed a CNN–BiGRU model for DNA sequence prediction, whereas Li et al. [31] introduced SIMEON, which employs stacked bidirectional GRU residual networks to identify chemical–protein interactions and capture higher‐order dependencies.

Although GRU‐based deep learning imputation methods generally outperform traditional statistical approaches in terms of accuracy and robustness [32], existing models often exhibit noticeable deviations from biological ground truth when applied to scRNA‐seq datasets with high dropout rates [33]. One key challenge lies in the inherently long and sparse nature of scRNA‐seq expression sequences, which makes it difficult for deep learning models to effectively capture long‐range dependencies, often leading to selective memory loss. Segmenting long sequences into shorter subsequences has been shown to enhance feature extraction and improve model performance.

Motivated by these observations, we propose a novel and efficient imputation framework termed imputation with a parallel gated recurrent unit neural network (IPGRU). The proposed IPGRU introduces two major improvements over existing imputation approaches. First, cyclic memory‐based hidden representations are leveraged to estimate the number of recoverable dropout genes and perform accurate value correction. Second, the original multifeature prediction task is decomposed into multiple single‐feature prediction problems through parallel segmentation, substantially reducing model complexity and improving learning efficiency. Unlike autoencoder‐based methods that rely on global latent representations, IPGRU decomposes the imputation problem into multiple localized prediction tasks, enabling more efficient learning of gene‐specific dependencies. This design improves the tractability of modeling high‐dimensional gene dependencies and enhances robustness in highly sparse data scenarios, although it introduces additional computational overhead. Extensive experiments on imputation accuracy and cell clustering demonstrate that IPGRU achieves state‐of‐the‐art performance across multiple scRNA‐seq datasets of varying sizes. The code is available on the GitHub website (github.com/zhao123minghao/IPGRU.git).

## 2 RESULTS

To validate the imputation results, we used three metrics to assess the methods—mean squared error on nonzero entries (MSE_NZ), mean squared error (MSE), and cluster score—and performed the downstream analysis. The experimental setup for this paper is as follows: a hardware platform with 24‐core Intel Xeon, 128 GB of memory, and an NVIDIA GeForce GTX 2080 Ti with 22 GB. The software platform is the Ubuntu 18.04 operating system, Python 3.7.4, and TensorFlow 1.13.1.

### 2.1 The datasets

We evaluated IPGRU and other methods using five public datasets, as shown in Table 1.

**TABLE 1:** Dataset description.

Dataset	Gene count	Cell count	Input dimension	GT	Nonzero rate
PBMC_G949_21K	949	21,065	900	Y	40.90%
GTEx_4tissues	56,202	3164	55,000	Y	49.80%
MAGIC_mouse	16,114	2576	15,900	Y	100.00%
PBMC_G949_10K	949	10,000	900	Y	33.00%
PBMC_G5561	5561	53,970	5400	N	9.90%

Abbreviation: GT, ground truth.

The PBMC_G949_21K [16, 34] dataset is a subsample of the 10x Genomics PBMC_G949 dataset. In our method, we focused on a set of 949 genes from 21,065 cells, with total read counts exceeding 10K (10,000) per gene and 15K (15,000) per cell. The dataset has a 41% nonzero rate, which is treated as ground truth directly. GTEx_4 tissues [35] are from the GTEx Portal V7 and contain gene expressions from 53 tissues. Here, we chose four tissues with a large sample size as ground truth, including muscle (564 samples), heart (600 samples), skin (1203 samples), and adipose tissue (797 samples), with a nonzero rate of 49.8%. Because GTEx_4 tissues are derived from bulk sequencing and this dataset contains more silent genes, we paid closer attention to MSE. MAGIC_mouse [17, 36] is a mouse bone marrow dataset. The original data are sparse, including hematopoietic stem cells at various stages of differentiation, which can reflect dynamics in gene expression. We generated an estimated matrix as the ground truth using the MAGIC method, randomly selected 30% of the ground truth for training, and compared the predicted matrix with the ground truth. PBMC_G949_10K is also generated from the PBMC_G949 dataset [34]. We selected the same 949 genes in the PBMC_G949 mentioned above and subsampled 10 K cells as ground truth. The nonzero rate of PBMC_G949_10K is 33%, and the dropout rate is set to 20%. PBMC_G5561 [34] is from 10x Genomics PBMC. It contains a set of 5561 genes selected from 21,065 cells, with counts per gene and per cell exceeding 1 K. The dataset has a 9.9% nonzero rate and is selected to evaluate the performance of our method under a high miss rate.

To validate the downstream analytical performance of various imputation methods, we selected three additional single‐cell datasets: human bone marrow [37], dentate gyrus neurogenesis [38], and pancreas [39]. The human bone marrow dataset comprises 5780 human bone marrow cells, profiled across 2000 genes after filtering. The dentate gyrus neurogenesis dataset contains 2930 cells and the same 2000 filtered genes, with a known differentiation trajectory from oligodendrocyte precursor‐like (OPL) cells to mature oligodendrocytes (OL). The pancreas dataset includes 2531 cells and 2000 filtered genes, encompassing multiple developmental lineages.

To address the intrinsic high sparsity of scRNA‐seq data, we developed a logistic regression‐based zero‐simulation framework that dynamically computes the probability of zeroing only nonzero expression values based on their magnitude. This approach preserves the biological significance of true zeros while allowing precise control over the proportion of introduced technical zeros. Let *x* denote the single‐cell count matrix, and let $x\in R^{+}$ represent a nonzero expression value. The probability of *x* being set to zero is then defined as follows:

(1) $$P_{\text{dropout}}\left(x\right)=\frac{1}{1+e^{-\left(\alpha +\beta \;\ln\left(x+1\right)\right)}}$$

where *α* is a global tuning hyperparameter that controls the overall dropout rate, whereas *β* is a dependency hyperparameter fixed at −1 in our experiments to reflect the lower dropout propensity of highly expressed genes. We applied a log‐transform to map expression values to probability space, establishing an inverse relationship between expression magnitude and dropout probability—consistent with higher‐abundance transcripts being more reliably detected. Three dropout regimes (20%, 30%, and 40%) were systematically evaluated to benchmark imputation performance across biologically relevant levels of missingness. For each target rate, we optimized the regularization parameter α through constrained bisection search.

### 2.2 Experimental results

We compared IPGRU with four methods: MAGIC [17], DeepImpute [19], AutoImpute [20], and DCA [32]. Table 2 compares MSE_NZ across methods on multiple datasets. IPGRU consistently achieves the lowest error rates in four datasets, particularly on GTEx_4tissues, where it achieves the lowest error of 0.033385. This suggests that IPGRU consistently achieves the lowest error rates, indicating superior imputation performance and adaptability across datasets. Other methods, such as AutoImpute, DeepImpute, and DCA, perform well but exhibit slightly higher error rates, whereas MAGIC shows variability and higher error rates on certain datasets, suggesting potential limitations in handling complex biological diversity.

**TABLE 2:** Comparison of MSE_NZ in all methods.

	PBMC_G949_21K	GTEx_4tissues	MAGIC_mouse	PBMC_G949_10K	PBMC_G5561
MAGIC	0.011913	2.376024	0.014339	0.011640	0.003866
DeepImpute	0.007827	0.052267	0.001575	0.006954	0.001304
AutoImpute	0.008430	0.076621	**0.001113**	0.007457	0.001362
DCA	0.012553	0.092522	0.002790	0.013850	0.001321
IPGRU	**0.006669**	**0.033385**	0.001503	**0.005850**	**0.001154**

*Note*: Bold text indicates the optimal value.

Abbreviation: MSE_NZ, mean squared error on nonzero entries.

Table 3 presents a comparison of MSE across the five datasets. From Table 3, we see that IPGRU offers state‐of‐the‐art accuracy, performing robustly even with high‐dimensional data. IPGRU consistently achieves the lowest error rates in four datasets, with standout performance on PBMC_G949_21K (0.007482) and PBMC_G949_10K (0.006923). Its ability to maintain minimal error across datasets, especially with complex data such as GTEx_4tissues (0.05437), suggests strong adaptability and effectiveness in imputation tasks. DeepImpute also performs well, with low error rates and competitive results, especially on simpler or synthetic datasets such as MAGIC_mouse. DCA and MAGIC provide moderate performance, with MAGIC showing significant variability, especially on GTEx_4 tissues. AutoImpute generally has higher error rates and exhibits limited adaptability, indicating that it may be less effective for high‐dimensional or complex data.

**TABLE 3:** Comparison of MSE in all methods.

	PBMC_G949_21K	GTEx_4tissues	MAGIC_mouse	PBMC_G949_10K	PBMC_G5561
MAGIC	0.05222	2.3760	0.01433	0.05543	0.01304
DeepImpute	0.008298	0.11328	0.002992	0.008473	0.001487
AutoImpute	0.08246	0.2450	0.003004	0.08666	0.09388
DCA	0.08114	0.1006	**0.002355**	0.08552	0.09256
IPGRU	**0.007482**	**0.05437**	0.003348	**0.006923**	**0.001342**

*Note*: Bold text indicates the optimal value.

Abbreviation: MSE, mean squared error.

Overall, IPGRU offers the best balance of accuracy and consistency in MSE and MSE_NZ. IPGRU performs slightly differently from DeepImpute and AutoImpute, yet achieves similar performance. Because MAGIC_mouse is a dataset synthesized by MAGIC, it introduces nonzero values into scRNA‐seq, which may lead to the failure to reconstruct the actual gene expression in single cells. In the imputation process, IPGRU could not distinguish whether a gene was expressed or generated by the MAGIC method, so IPGRU may be inferior to other methods in these two results.

Furthermore, we conducted extensive experiments on datasets for cell trajectory inference analysis across varying dropout rates, with results presented in Table 4. Across all three datasets and dropout levels, IPGRU consistently matches or outperforms competing methods in reconstructing nonzero entries. On human bone marrow, IPGRU achieves MSE_NZ of 0.000948, 0.000952, and 0.000959 at 20%, 30%, and 40% dropout, respectively—surpassing MAGIC by roughly threefold to fourfold and edging out DeepImpute at higher dropout rates. IPGRU outperformed MAGIC, DeepImpute, and AutoImpute across the most dropout levels and outperformed DCA at 20% and 40% dropout. On the pancreas dataset, IPGRU tied with DeepImpute for the lowest MSE_NZ at 20% dropout and achieved the lowest errors at 30% and 40% dropout. It also consistently outperformed AutoImpute, DCA, and MAGIC across all dropout levels. Collectively, these results demonstrate that IPGRU maintains robust and consistently competitive imputation accuracy as the proportion of missing data increases.

**TABLE 4:** Comparison of MSE_NZ in all methods across dropout rates.

	Human bone marrow			Dentate gyrus neurogenesis			Pancreas		
	20%	30%	40%	20%	30%	40%	20%	30%	40%
MAGIC	0.003170	0.003410	0.003410	0.004462	0.004776	0.005165	0.004655	0.004998	0.005414
DeepImpute	**0.000930**	**0.000912**	0.000985	0.001385	0.001500	0.001559	**0.001251**	0.001240	0.001251
AutoImpute	0.001016	0.001013	**0.000959**	0.001431	0.001421	0.001425	0.001312	0.001306	0.001333
DCA	0.000965	0.000991	0.001020	0.001488	**0.001357**	0.001412	0.001541	0.001407	0.001438
IPGRU	0.000948	0.000952	**0.000959**	**0.001357**	0.001370	**0.001382**	**0.001251**	**0.001224**	**0.001246**

*Note*: Bold text indicates the optimal value.

Abbreviation: MSE_NZ, mean squared error on nonzero entries.

Figures 1 and 2 illustrate the comparative performance across three biological datasets (human bone marrow, dentate gyrus neurogenesis, and pancreas) through a series of boxplots. Each row in Figure 1 depicts results from a single dataset under varying dropout rates (20%, 30%, and 40%), with technical error quantified using MSE_NZ. Each row in Figure 2 shows biological consistency, evaluated using Pearson correlation coefficients for both cell–cell and gene–gene relationships. The results demonstrate that IPGRU consistently achieves the lowest error distributions and the highest correlation coefficients across all dropout scenarios, underscoring its effectiveness in enhancing data quality through imputation.

**FIGURE 1:**
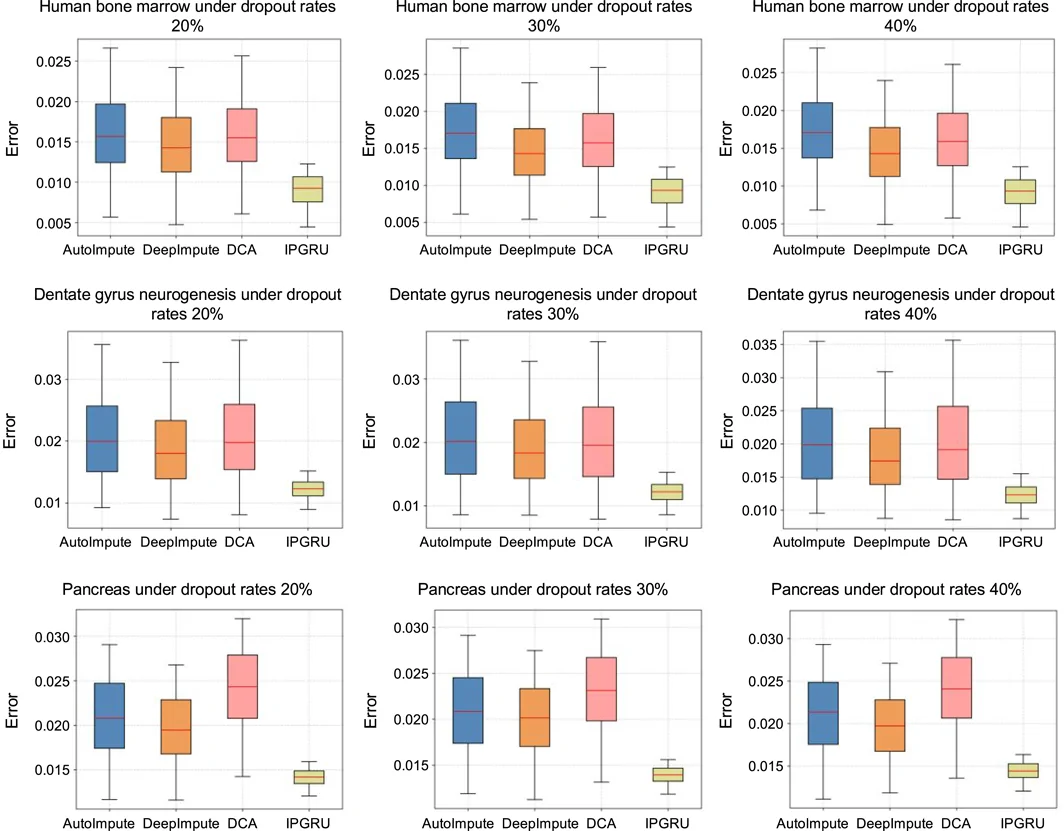
The boxplots of imputation errors across varying dropout rates (20%–40%).

**FIGURE 2:**
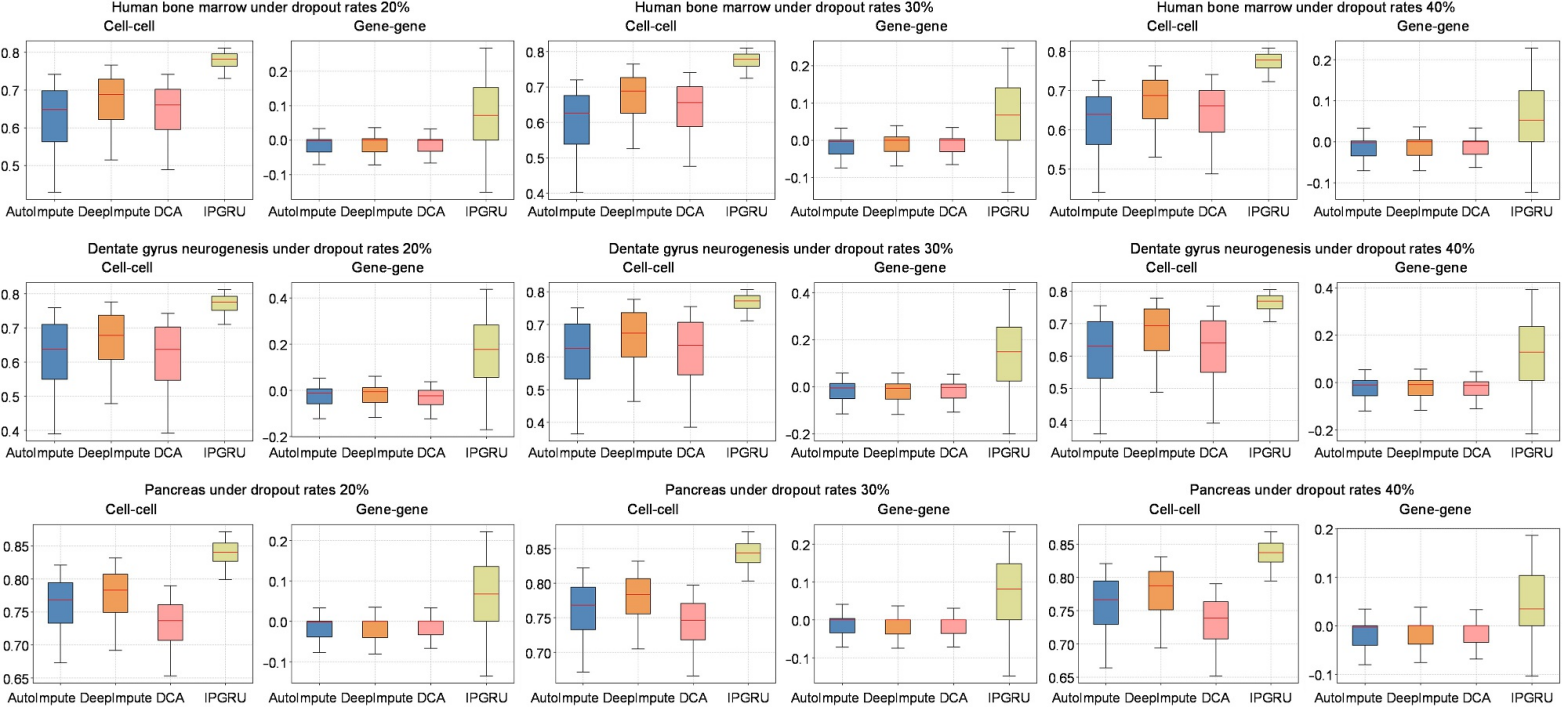
The boxplots of cell–cell and gene–gene similarities across varying dropout rates (20%–40%).

### 2.3 Comparison of downstream analysis results

scRNA‐seq datasets typically consist of heterogeneous mixtures of cell types, and high dropout rates severely obscure transcriptional differences among cells. To evaluate the impact of imputation on downstream clustering performance, we conducted clustering analyses on three annotated scRNA‐seq datasets: human bone marrow [37], dentate gyrus neurogenesis [38], and pancreas [39].

We assessed clustering performance under a challenging condition with a simulated 40% dropout rate. In raw scRNA‐seq data, sparse expression matrices are dominated by numerous false‐zero values, which substantially impair dimensionality reduction and clustering by masking true transcriptional signals. For consistency, Uniform manifold approximation and projection (UMAP) [40] was applied across all experiments to reconstruct the low‐dimensional manifold structure of cell populations from high‐dimensional gene expression data.

As shown in Figure 3, the first column shows clustering results from the raw input data. Cells exhibit highly intermingled distributions with fragmented and overlapping clusters, indicating poor separability of cell types. In particular, closely related populations such as hematopoietic stem cell subpopulations (HSC_1 and HSC_2) in the human bone marrow dataset lack clear boundaries, a direct consequence of global sparsity induced by technical dropout events. In contrast, clustering results after IPGRU imputation (rightmost column) reveal well‐defined and compact clusters across all three datasets, with clear separation between major cell populations.

**FIGURE 3:**
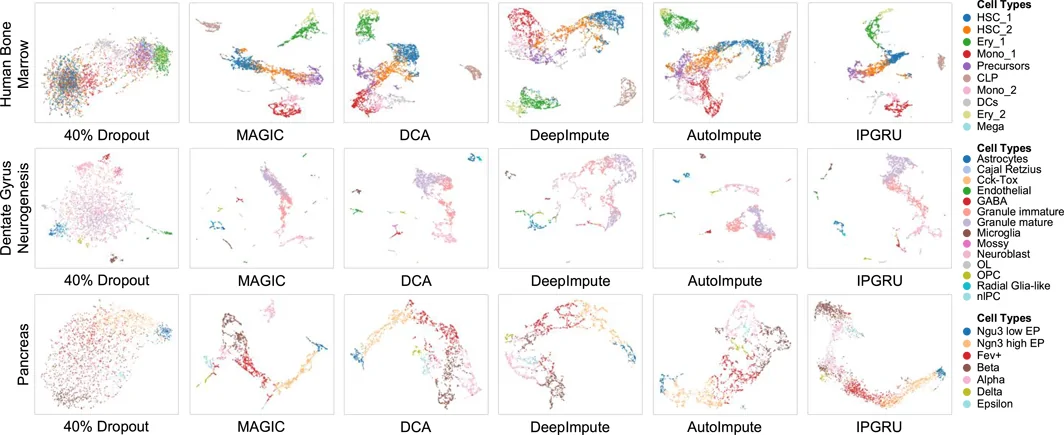
Cluster visualizations across different imputation methods at 40% dropout. Cck‐Tox, Cck‐ and Tox‐expressing neuronal cells; CLP, common lymphoid progenitor cells; DCs, dendritic cells; EP, endocrine progenitor cells; Ery, erythroid cells; Fev, Fev‐expressing serotonergic neuronal cells; GABA, GABAergic neurons; HSC, hematopoietic stem cells; Mega, megakaryocytes; Mono, monocytes; Ngn3, neurogenin 3‐expressing endocrine progenitor cells; nIPC, neural intermediate progenitor cells; OL, oligodendrocytes; OPC, oligodendrocyte precursor cells.

We further compared IPGRU with MAGIC, DCA, DeepImpute, and AutoImpute. All imputation methods improved clustering performance relative to raw data, demonstrating enhanced discrimination of cell types. However, IPGRU consistently achieved the most accurate clustering recovery across all datasets. In the human bone marrow dataset, IPGRU distinctly separated hematopoietic stem cell subpopulations (HSC_1 and HSC_2) into compact, nonoverlapping clusters and clearly distinguished erythroid populations (Ery_1 and Ery_2) from myeloid lineages (Mono_1, Mono_2, DCs, Mega, etc.). Moreover, the spatial organization of these clusters faithfully reflected the known hematopoietic differentiation hierarchy from stem cells toward erythroid and myeloid lineages.

For the pancreas dataset, IPGRU produced highly consistent clustering of endocrine cell populations, including beta, alpha, delta, and epsilon cells. The resulting UMAP embeddings exhibited smooth gradient‐like transitions that align with established developmental relationships. In contrast, MAGIC incorrectly subdivided the alpha cell population into two distinct clusters, suggesting oversmoothing artifacts.

DeepImpute and AutoImpute demonstrated moderate performance on the human bone marrow and pancreas datasets. Although major cell types were partially separated, cluster boundaries remained ambiguous, and overlaps persisted, particularly for rare cell populations. DCA showed relatively conservative imputation behavior. Although it reduced noise to some extent, it failed to fully resolve intracluster mixing caused by dropout. MAGIC’s diffusion‐based smoothing occasionally led to overdispersion, most notably in the dentate gyrus neurogenesis dataset, where the embedding fragmented into multiple small, disconnected subclusters. This fragmentation disrupted biologically expected continuous developmental trajectories and compromised downstream lineage inference.

Figure 4A presents differentiation trajectories inferred using the stochastic scVelo model [41] on the human bone marrow dataset, comparing raw data with 40% dropout (left) to data imputed by IPGRU (right). Trajectories derived from dropout‐affected data display blurred branching structures and misaligned velocity vectors, reflecting technical noise. In contrast, IPGRU‐imputed data recover smooth and continuous differentiation pathways. The enlarged view highlights that velocity vectors at branching points exhibit clear directional consistency after imputation, whereas vectors derived from raw data lack coherent orientation.

**FIGURE 4:**
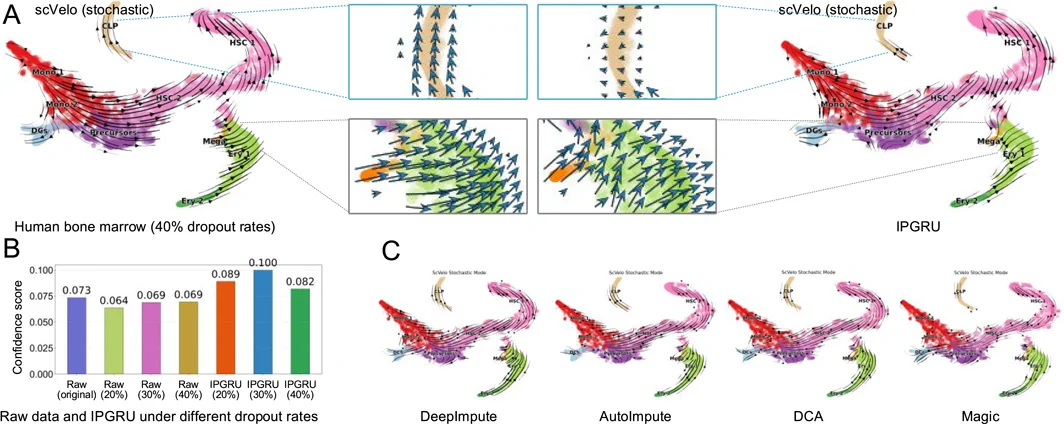
Comparative analysis of imputation impact on single‐cell differentiation dynamics. (A) Trajectory inference on the human bone marrow dataset: comparison between original data with 40% dropout and data imputed by IPGRU. (B) Quantitative evaluation using the cross‐boundary confidence score. (C) Comparison of trajectory inference results on data imputed by various methods. CLP, common lymphoid progenitor cells; DCs, dendritic cells; Ery, erythroid cells; HSC, hematopoietic stem cells; Mega, megakaryocytes; Mono, monocytes.

To quantitatively assess trajectory reconstruction reliability, we employed the cross‐boundary confidence score [42], which measures directed transition strength between source and target clusters based on RNA velocity probabilities. As shown in Figure 4B, IPGRU‐imputed data achieve consistently higher confidence scores than raw inputs across 20%, 30%, and 40% dropout rates and even outperform the fully raw dataset without simulated dropout. These results indicate that IPGRU not only compensates for technical missingness but also corrects systematic biases, thereby enhancing biologically meaningful signals. Figure 4C further compares trajectory inference results obtained using DeepImpute, AutoImpute, DCA, and MAGIC under identical conditions. IPGRU produces the most coherent and biologically plausible branching structures, confirming its superior capability to denoise sparse scRNA‐seq data and improve downstream lineage inference accuracy.

To evaluate computational efficiency, we compared the number of model parameters, memory usage, and inference time across different methods. As shown in Table 5, the overall memory usage is similar across all methods (approximately 2.34–2.38 GB). This is mainly because the measurements reflect the total runtime memory consumption of the process, including shared components such as the deep learning framework and data loading pipeline, rather than the model parameters alone. In contrast, differences in model complexity are more clearly reflected in the number of parameters and inference time. AutoImpute and DeepImpute contain 122,010 and 160,010 parameters, respectively, both with an average inference time of 5 ms. DCA has a relatively lightweight structure with 8106 parameters and an inference time of 6 ms. In contrast, IPGRU contains 788,780 parameters and requires approximately 14 ms for inference.

**TABLE 5:** Comparison of model complexity and inference time.

	Parameters	Memory usage (MB)	Inference time (ms)
DeepImpute	160,010	2341.77	5
AutoImpute	122,010	2356.64	5
DCA	8106	2357.43	6
IPGRU	788,780	2380.12	14

## 3 DISCUSSION

The parallel GRU neural network proposed in this study implicitly learns gene–gene dependencies during prediction, yielding a gene correlation coefficient matrix that captures similarities among gene expression patterns. This representation enables the identification of highly correlated gene groups and facilitates the accurate imputation of missing gene expression values. By modeling nonlinear gene dependencies, the proposed framework helps restore disrupted gene relationships and supports the reconstruction of biologically meaningful gene coexpression networks.

Unlike existing imputation approaches, IPGRU introduces a task‐decomposition strategy that segments the original multifeature prediction problem into multiple independent single‐feature prediction subnetworks. This parallelized architecture substantially reduces model complexity and improves computational efficiency when handling high‐dimensional scRNA‐seq data. By exploiting the dependency structures learned within each GRU module, dropout events are effectively identified and corrected through recurrent modeling, thereby mitigating the impact of outliers and technical noise in scRNA‐seq datasets.

Overall, IPGRU performs well in accurately imputing missing values in scRNA‐seq data. The memory mechanisms inherent to GRU units enable the model to capture complex gene expression dynamics, whereas backpropagation progressively refines network parameters to enhance forward prediction accuracy. As demonstrated in our experiments, IPGRU achieves state‐of‐the‐art or comparable performance across multiple benchmark scRNA‐seq datasets.

Despite these advantages, IPGRU also has certain limitations. GRU‐based architectures are computationally more demanding than convolutional neural networks, and although GRUs are generally more efficient than LSTM‐based models, nonlinear activation functions such as sigmoid and tanh introduce additional computational overhead. Although the model leverages gene–gene dependencies to mitigate overimputation, distinguishing technical dropouts from biologically meaningful zeros remains a challenging problem. Although this study focuses on comparing IPGRU with representative statistical and neural network‐based imputation methods, GNN‐based approaches such as scGNN have shown strong performance in modeling cell–cell relationships. Because of differences in modeling paradigms and implementation complexity, such methods were not included in the current benchmark. A comprehensive comparison with GNN‐based methods and potential integration of graph‐based and recurrent architectures will be explored in future work.

## 4 CONCLUSIONS

In this study, we developed IPGRU, a parallel gated recurrent unit neural network for imputing dropout events in scRNA‐seq data. By decomposing the original multifeature prediction problem into multiple parallel single‐feature prediction tasks, IPGRU efficiently models gene‐specific dependencies while reducing the complexity of high‐dimensional imputation.

Across multiple benchmark datasets, IPGRU achieved consistently low MSE and MSE_NZ values and demonstrated competitive or superior performance compared with MAGIC, DeepImpute, AutoImpute, and DCA. In addition, evaluations under different dropout rates showed that IPGRU maintained robust imputation accuracy as data sparsity increased. The imputed datasets also improved downstream cell clustering and differentiation trajectory inference, generating more clearly separated cell populations and biologically coherent lineage structures.

Overall, IPGRU provides an effective and robust framework for recovering missing expression signals in scRNA‐seq data and improving the reliability of downstream biological analyses. Nevertheless, the model has a higher number of parameters and longer inference time than several competing methods, and distinguishing technical dropouts from biologically meaningful zeros remains challenging. Future work will focus on improving computational efficiency, enhancing model interpretability, and integrating recurrent architectures with graph‐based methods to better capture cell–cell and gene–gene relationships.

## 5 MATERIALS AND METHODS

The structure of IPGRU and the process of scRNA‐seq imputation are shown in Figure 5. The whole process includes preprocessing, network building, training, and evaluation metrics.

**FIGURE 5:**
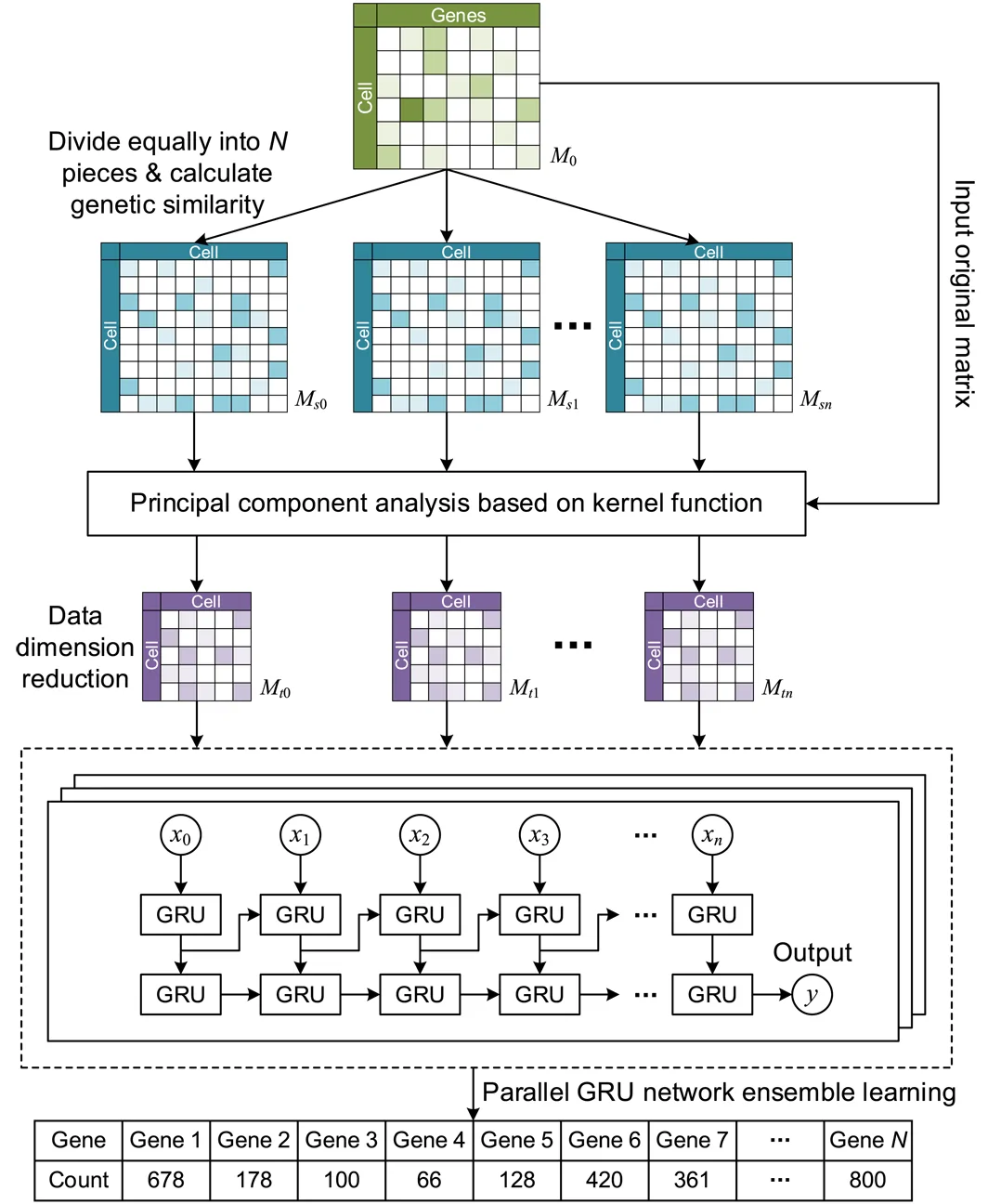
Process of scRNA‐seq imputation (IPGRU).

### 5.1 Preprocessing

The IPGRU method employs a divide‐and‐conquer strategy during deep learning inference. We split the dataset into *N* random subsets, each with *S* genes, called “target genes.” By default, *S* is set to 300. Based on the gene expression matrix, the top 5 genes most related to the target genes are set as the predicted genes.

### 5.2 Imputation model

The scRNA‐seq data imputation method includes data preprocessing, dimensionality reduction, and ensemble learning using parallel GRU networks. The process is shown in Figure 5.

The imputation model begins with data preprocessing. The scRNA‐seq count matrix is a substantial two‐dimensional matrix with cell types on the horizontal axis [33] and genes on the vertical axis. As the numbers in the count matrix are distributed, we normalize the matrix. Then, gene similarity analysis is performed, primarily using the Pearson correlation coefficient, defined as follows:

(2) $$\rho \left(X,Y\right)=\frac{E\left[\left(X-\mu x\right)\left(Y-\mu y\right)\right]}{\sigma_{X}\sigma_{Y}}$$

In Equation (2), *X* and *Y* are two gene tags. The Pearson correlation coefficient is a measure of linear correlation between two variables, equal to 1 or −1 when the data are distributed along a straight line. The count matrix is converted to a gene correlation coefficient matrix using the Pearson correlation coefficient. Finally, the original cell count matrix is randomly split into groups of 300, forming the subdatasets, as shown in Figure 5. Specifically, the choice of subset size (*S* = 300) and the number of top correlated genes (5) are based on an empirical trade‐off between computational efficiency and imputation accuracy. A moderate subset size allows sufficient modeling of gene dependencies while avoiding excessive computational cost. Similarly, selecting the top 5 correlated genes provides meaningful contextual information without introducing noise from weak correlations.

The kernel‐based principal component analysis algorithm is used to select the top 5 most relevant genes for the target gene from the correlation coefficient matrix, forming the predicted gene matrix. The predicted gene matrix is fed into multiple sub‐recurrent neural networks (sub‐GRUs). The genes were provided as branches, and parallel and distributed computing was used to speed up model training. Each sub‐recurrent neural network aims to understand the relationship between the input and target genes.

The IPGRU is used to predict missing values in scRNA‐seq data, and the gated RNN architecture is shown in Figure 6. The GRU is a variant of the RNN that adds a gate to control the transmission of information and avoid gradient disappearance and gradient explosion, thereby meeting the characteristics of scRNA‐seq data and predicting unknown gene dependencies.

**FIGURE 6:**
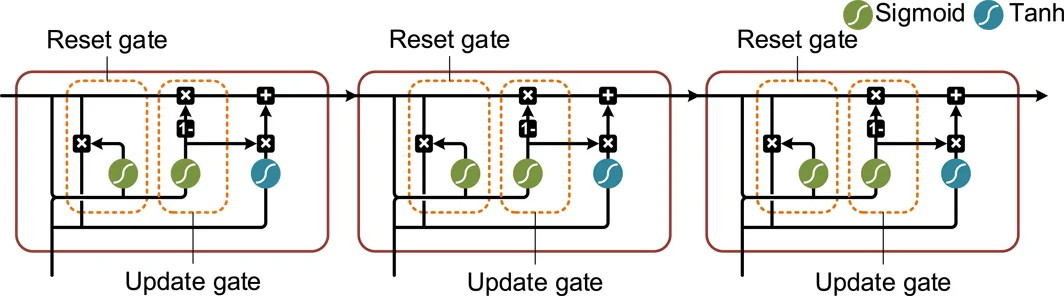
Architecture of a gated recurrent unit (GRU).

The GRU neural network controls memory and updates its weights via the reset and update gates. The following equation defines the reset gate:

(3) $$r_{t}=\sigma \left(W_{r}\cdot \left[h_{t-1},x_{t}\right]\right),$$

where *r*_*t*_ is the vector for the reset gate, which is responsible for memorizing the current state. *W*_*r*_ is the reset gate weight matrix, *h*_*t−1*_ is the state vector at time *t*−1, and *x*_*t*_ is the scRNA‐seq data input value at time *t*. The update gate works as follows:

(4) $$z_{t}=\sigma \left(W_{z}\cdot \left[h_{t-1},x_{t}\right]\right),$$

where *z*_*t*_ is the update gate vector, *W*_*z*_ is the weight matrix of the update gate, and *σ* stands for the sigmoid activation function.

(5) $$\begin{array}{l} {\tilde{h}}_{t}=\tanh\left(W\cdot \left[r_{t}\cdot h_{t-1},x_{t}\right]\right)\\ h_{t}=\left(1-z_{t}\right)\cdot h_{t-1}+z_{t}\cdot {\tilde{h}}_{t} \end{array},$$

where ${\tilde{h}}_{t}$ stands for the candidate state vector and $h_{t}$ stands for the output vector. The reduced‐dimensional data are input to multiple groups of GRU neural networks, and the parallel networks predict candidate sequences. Finally, we obtained the imputed results from the fully connected neural network ensemble. In Figure 6, the counts for genes 3 and 6 (“793” and “420”) are the imputed values generated by the imputation model, which are zero in the original datasets. Nonbolded numbers in the result of Figure 5 are not dropouts, and the imputation model retained their original values. The IPGRU model can find zero values (dropouts) and correct them without destroying the original correct data.

### 5.3 Network building and training

In the conquer step, we trained some GRU neural networks. In the single network, the first layer is the input layer. There are 768 GRUs in the second‐layer network and 640 GRUs in the third‐layer network. A fully connected layer network with 300 neurons is connected to the third layer. A dropout is placed after each GRU layer, with a dropout rate of 0.5 to prevent overfitting on the training set.

The optimizer for training is Adam. The learning rate is 0.001, the batch size is 32, the number of genes per subset is 300, and the number of epochs is 100. If the model’s validation loss does not decrease within 3 epochs of training, the subnetwork training is stopped early.

The training data are split into training (90%) and test (10%) sets. The test sets are used to evaluate the network accuracy after each epoch. We use MSE_NZ as the loss function for the entire model, which emphasizes the accuracy of high‐confidence values and avoids errors for genes with extremely low values. The loss is defined as follows:

(6) $${\text{MSE}}_{\text{NZ}}=\frac{\sum_{i}^{m}\sum_{j}^{n}\left(Y_{ij}-T_{ij}\right)I\left(X_{ij}\neq 0\right)}{\sum_{i}^{m}\sum_{i=1}^{n}I\left(X_{ij}\neq 0\right)}.$$

where *m* represents the number of rows in the prediction matrix, *n* is the number of columns in the prediction matrix, *Y*_*ij*_ is the predicted gene value, and *T*_*ij*_ is the ground truth value. *I* is a function. *I* = 1 when the count matrix is traversed to a value that is not zero. In contrast, *I* = 0 when the count matrix is traversed to zero for a specific value. MSE_NZ could evaluate the differences between the nonzero values of the prediction matrix and ground truth.

### 5.4 The evaluation index

To validate the results, we used three metrics to evaluate the methods: MSE_NZ, MSE, and cluster score. The MSE for all values of the predicted and real matrices is calculated as follows:

(7) $$\text{MSE}=\frac{\sum_{i}^{m}\sum_{j}^{n}{\left(Y_{ij}-T_{ij}\right)}^{2}}{mn},$$

where *m* represents the number of rows in the prediction matrix, *n* stands for the number of columns in the prediction matrix, *Y*_*ij*_ is the generated predicted gene value, and *T*_*ij*_ is the ground truth gene value.

Then, in the downstream analysis, we used the Calinski–Harabasz score to evaluate clustering performance, which can indirectly assess the correctness of the dropout values. A set *E* of size *n*_*E*_ has been clustered into *k* clusters. The Calinski–Harabasz score *s* is defined as the ratio of the intercluster dispersion mean to the intracluster dispersion mean:

(8) $$s=\frac{tr\left(B_{k}\right)}{tr\left(W_{k}\right)}\times \frac{n_{E}-k}{k-1},$$

(9) $$W_{k}=\sum_{q=1}^{k}\sum_{x\in C_{q}}\left(x-c_{q}\right){\left(x-c_{q}\right)}^{T},$$

(10) $$B_{k}=\sum_{q=1}^{k}n_{q}\left(c_{q}-c_{E}\right){\left(c_{q}-c_{E}\right)}^{T},$$

where tr(*B*_*k*_) is the trace of the between‐group dispersion matrix, and tr(*W*_*k*_) is the trace of the within‐cluster dispersion matrix. *C*_*q*_ is the set of points in cluster *q*, *c*_*q*_ is the center of cluster *C*_*q*_, *c*_*E*_ is the center of *E*, and *n*_*q*_ is the number of points in cluster *q*.

## AUTHOR CONTRIBUTIONS

**Shuang Xu:** Conceptualization; methodology; software; investigation; formal analysis; data curation; visualization; writing—original draft. **Xiangtao Li:** Conceptualization; methodology; validation; writing—review and editing. **Jingsong Li:** Software; validation; data curation; writing—review and editing. **Yu Jiang:** Conceptualization; supervision; project administration; writing—review and editing. All authors have read and approved the final manuscript.

## CONFLICT OF INTEREST STATEMENT

The authors declare no conflicts of interest.

## ETHICS STATEMENT

The authors declare that the study does not include animal and human experiments that violate ethics. This study is a computational analysis based solely on publicly available datasets and does not involve the collection of new human or animal data.

## Data Availability

All data in this article are available. Mouse bone marrow data: GEO GSE72857 (ncbi.nlm.nih.gov/geo/query/acc.cgi?acc=GSE72857). GTEx gene expression data: GTEx (gtexportal.org/home/datasets). Human PBMC from 10x Genomics: 10x Genomics (support.10xgenomics.com/single‐cell‐gene‐expression/datasets). Human bone marrow: Human cell atlas (data.humancellatlas.org/explore/projects/091cf39b‐01bc‐42e5‐9437‐f419a66c8a45). Dentate gyrus neurogenesis: GEO GSE95753 (ncbi.nlm.nih.gov/geo/query/acc.cgi?acc=GSE95753). Pancreas: GEO GSE132188 (ncbi.nlm.nih.gov/geo/query/acc.cgi?acc=GSE132188).
